# Different effects of paternal trans-generational immune priming on survival and immunity in step and genetic offspring

**DOI:** 10.1098/rspb.2014.2089

**Published:** 2014-12-22

**Authors:** Hendrik Eggert, Joachim Kurtz, Maike F. Diddens-de Buhr

**Affiliations:** Institute for Evolution and Biodiversity, Westfälische Wilhelms-Universität Münster, Hüfferstraße 1, Münster 48149, Germany

**Keywords:** insect immunity, paternal immune priming, transgenerational immune priming, *Tribolium castaneum*, *Bacillus thuringiensis*

## Abstract

Paternal trans-generational immune priming, whereby fathers provide immune protection to offspring, has been demonstrated in the red flour beetle *Tribolium castaneum* exposed to the insect pathogen *Bacillus thuringiensis*. It is currently unclear how such protection is transferred, as in contrast to mothers, fathers do not directly provide offspring with a large amount of substances. In addition to sperm, male flour beetles transfer seminal fluids in a spermatophore to females during copulation. Depending on whether paternal trans-generational immune priming is mediated by sperm or seminal fluids, it is expected to either affect only the genetic offspring of a male, or also their step offspring that are sired by another male. We therefore conducted a double-mating experiment and found that only the genetic offspring of an immune primed male show enhanced survival upon bacterial challenge, while phenoloxidase activity, an important insect immune trait, and the expression of the immune receptor PGRP were increased in all offspring. This indicates that information leading to enhanced survival upon pathogen exposure is transferred via sperm, and thus potentially constitutes an epigenetic effect, whereas substances transferred with the seminal fluid could have an additional influence on offspring immune traits and immunological alertness.

## 1. Introduction

Trans-generational immune priming is a phenomenon describing the transfer of immune stimulation from the parental to the offspring generation. This transfer of immunity is known for vertebrates [1–5], invertebrates [6–11] and also plants [12], although the underlying mechanisms are diverse.

Characteristic for vertebrates is the maternal transfer of immunity, where mothers provide their offspring with hormones, nutrients and antibodies that enhance the offspring fitness [1–4]. In mammals, maternal factors can be transferred via the placenta and breast milk during lactation [13,14], whereas in birds, reptiles and fishes, they are mainly transferred via the egg [4,15,16]. Exceptional cases are sex-role-reversed species like pipefish, seahorses and sea dragons which provide a unique opportunity to test for adaptive plasticity in parental immune transfer. Here, males and females both influence offspring immunity [17]. Paternal effects influencing the immune system of offspring are rarely documented in vertebrates [18], however, there is growing evidence in insects [6–8,11,19–24]. In some insects only females transferred immunity [6,7,10,23,25], whereas in others, males also are able to induce protection in the next generation [11,26] and yet some insect species did not show the effect at all [20,22]. Paternal immune stimulation with heat-killed bacteria or lipopolysaccharides was shown to enhance several immune effectors and to prime the offspring in a more general way than maternal immune priming. This was shown first in the red flour beetle, *Tribolium castaneum* [26] and thereafter in the mealworm beetle, *Tenebrio molitor* [23]. Triggs & Knell [27] could show for Lepidoptera that paternal diet has strong trans-generational effects on offspring immunity. Unfortunately, little is known about the underlying mechanisms.

Paternal immune priming might be mediated through genetic imprinting or through the transfer of modifying factors inside sperm or in the seminal fluid [28,29]. It has the potential to strongly influence the epidemiology and evolution of host–parasite interactions [30]. Using the red flour beetle, *T. castaneum*, and *Bacillus thuringiensis* (*Bt*), we investigated the transfer of paternal immune priming in a double-mating design. Hence, one female was allowed to sequentially mate with two males, one of which was primed, the other left naive. During copulation, male beetles invaginate a tube that everts into a sperm containing sac, a spermatophore, containing sperm and seminal fluids [31,32]. To answer the question of how the information is transferred via sperm or seminal fluid, we compared survival and constitutive phenoloxidase (PO) levels, as well as constitutive gene expression of offspring that were either related to the primed male or unrelated.

## 2. Material and methods

### (a) The model system

Owing to its small size, short generation time and feasibility of handling the model system, *T. castaneum* is very suitable to investigate ecology, behaviour and immunology of host–parasite interactions. As its genome sequence has been available since 2008, the red flour beetle facilitates gene function analysis and allows efficient genetic screens [33]. As a primarily grain dwelling organism and therefore major pest of stored cereals, *Tribolium* spp. are found worldwide [34]. Juveniles and adults live together in aggregations. Adults are long lived and females lay eggs continuously over their life [35]. Naturally, *T. castaneum* harbours a range of protozoans and other parasites [36–38]. In our experiment, we used *Bt* (strain DSM no. 2046) as a micro parasite, which was obtained from the German Collection of Microorganisms and Cell Cultures (DSMZ). *Bt* is an insect-specific pathogen, which was isolated from the Indian meal moth, *Plodia interpunctella*, and is known to infect *T. castaneum* [39,40].

Two populations of beetles were used in the double-mating design. The reindeer population (Rd) shows a dominant mutation in antennae morphology, resulting in exaggeration of an antennal club (in the shape of reindeer antlers) [41]. We received the strain from the Gage Laboratory (Centre for Ecology, Evolution and Conservation, University of East Anglia, Norwich, UK), originally cultured in the Beeman Laboratory (United States Department of Agriculture, Biological Research Unit, Grain Marketing and Production Research Center, Manhattan, KS, USA). The Croatia 1 (Cro1) population was collected in 2010 in Croatia [40] and since then kept under standard conditions in our laboratory (30°C, 70% humidity and a 12 L : 12 D cycle).

### (b) The experiment

**Figure 1.**
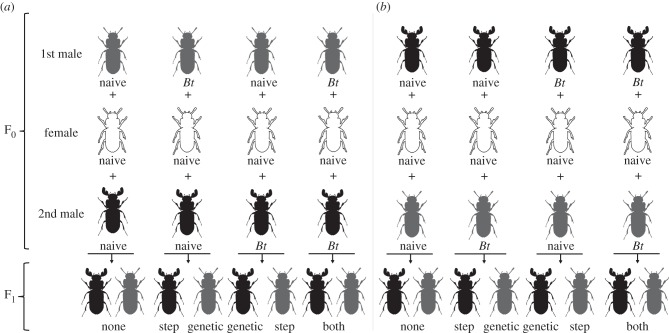
Double-mating design. To elucidate the way of transfer of paternal immune priming, we compare survival and immunity of step and genetic offspring of primed males. Each double-mating set consisted of a Cro1 female (white), an Rd male (black) and a Cro1 male (grey). To determine paternity, we used antenna morphology. The Rd males show a dominant mutation in the shape of reindeer antlers. Four priming treatments were established: (i) both males naive, (ii) Cro1 male primed, (iii) Rd male primed, (iv) both males primed. Treatment (i) served as the control, treatments (ii) and (iii) to exclude strain effects, and treatment (iv) to test for additive effects of paternal immune priming. To control for the effect of mating order, once Cro1 males mated first and Rd males mated secondly (*a*), and vice versa (*b*).

The idea was to compare survival and immunity of step and genetic offspring of primed *T. castaneum* males to find out how the information of paternal immune priming is transferred to the next generation. Therefore, a female was mated with two males, one primed and the other one naive (figure 1). To determine paternity, we used antenna morphology. The Rd males show a dominant mutation in the shape of reindeer antlers. Each double-mating set consisted of a Cro1 female, an Rd male and a Cro1 male. To test for the way of transfer of paternal immune priming we established four priming treatments: (i) both males naive, (ii) Cro1 male primed, (iii) Rd male primed, and (iv) both males primed. Treatment (i) served as the control, treatments (ii) and (iii) to exclude strain effects, and treatment (iv) to test for additive effects of paternal immune priming. In a former study, we proved that sham-treated parents behave similarly to naive parents and do not transfer resistance in terms of trans-generational priming [26]. Therefore, for feasibility reasons in this already extensive experiment, we did not include a sham treatment. To control for the effect of mating order once Cro1 males mated first and Rd males mated second and vice versa. The females were allowed to mate with each male for 24 h. For each treatment, we established 20 double-mating sets.

For the experiment, eggs of *T. castaneum* were individually distributed into 96-well plates, filled with flour and 5% yeast. Animals were raised at 30°C, 70% humidity and a 12 L : 12 D cycle. Individuals were checked regularly for their developmental stage. When the pupal stage was reached, animals were sexed and distributed individually into a fresh 96-well plate. Six weeks after the distribution of eggs all individuals had reached sexual maturity.

Male beetles were randomly assigned to one of two immune priming treatments 24 h before mating: naive or pricking with *Bt.* The males in the *Bt*-group were pricked between caput and thorax with a needle dipped in a phosphate-buffered saline (PBS) solution with 10^9^ bacteria per millilitre of heat-killed (30 min, 90°C) bacteria obtained from an overnight culture (for details see [42]). For each double-mating set-up the female and male were kept in a vial containing 3 g of flour with 5% yeast. After the second mating, the female was transferred to a new vial and allowed to lay eggs for 10 days. Every second day, each female was transferred to a new vial, resulting in five batches of offspring. Offspring were counted and individually distributed to 96-well plates. All offspring had grown to adults 35 days later and paternity was determined. For analysis of survival of phenoloxidase and gene expression, we used only offspring of the first two batches, considering that Zanchi *et al.* [11] demonstrated that only the early offspring showed immune priming in *T. molitor*. To evaluate fecundity all batches of offspring were counted.

### (c) Assays

Paternity of all offspring was examined by checking the antennae for the reindeer antler shape. According to the order of mating the offspring was assigned to the first or second male. The number of offspring of each batch was counted for each pair. The proportion of offspring sired by the second male (P_2_) was calculated.

Offspring survival after a bacterial challenge was measured (in days post-challenge) as a phenotypic outcome of paternal immune priming. From each father, three offspring were randomly chosen and assigned to one of three challenge treatments (naive, PBS, *Bt*). Challenge was performed with live bacteria, which were grown as described in Roth & Kurtz [42] and adjusted to a cell concentration of 10^11^ ml^−1^ in PBS solution. Animals in the sterile PBS group were treated accordingly, but pricked only with PBS solution. Thereafter, every beetle was randomly and individually distributed to 96-well plates filled with flour and 5% yeast. Survival after challenge was checked on days 1, 2, 3, 5 and 7. For each priming treatment, 40 individuals per challenge treatment were tested.

The activity of PO (*V*_max_), a key enzyme in insect immunity, in the haemolymph (i.e. the insects’ blood) was measured [43]. For this, the haemolymph of three offspring of one father was pooled into one sample. For each priming treatment, 48 pools of haemolymph were prepared. Four pools had been excluded owing to handling errors.

The haemolymph was collected by puncturing the pleural membrane between pronotum and occiput with a sterile hypodermic needle. Pools of haemolymph were handled and measured as described in Roth & Kurtz [42].

The expression of genes was measured by reverse transcription real-time PCR. We used 14 genes, including two housekeeping genes (table 1). To cover immune related expression, we looked at the immune deficiency pathways (Imd) and Toll pathway, by measuring the expression of the activating recognition proteins (*PGRP*, *GNBP*), the key genes *imd* and *toll* as well as the according antimicrobial peptides (*attacin*, *coleoptericin*, *thaumatin*). Furthermore, we addressed the gene *nimB*, as this protein is plasmatocyte-specific and participates directly in the phagocytosis of bacteria. We also included *lysozyme*, because lysozymes are ubiquitous components of innate immune response of insects. The enzyme is normally present in the haemolymph, and together with other bactericidal factors lysozyme is often strongly induced when the insect is infected. As we measured PO, we connected our physiological results with the expression of *proPO*. The proPO is an inactive zymogen, which is activated by proteolytic cleavage in the PO cascade to active PO, playing an important role in cuticular sclerotization and in defence against pathogens and parasites [44–47]. *Hsp90* and *hsp68* were used to detect differential expression upon stress [44,45]. We used 12 replicates per priming treatment. Each replicate is a pool consisting of eight beetles. Replicates were frozen in liquid nitrogen. Frozen beetles were lysed in liquid nitrogen with a sterile pestle and 500 µl of Trizol (Ambion RNA by Life Technologies GmbH, Darmstadt, Germany) were added to each sample. Samples were further lysed by incubation at room temperature (RT) for 10 min and mixed vigorously every 2 min. After centrifugation (18 000*g* at 4°C, 5 min), the supernatant was transferred to a new tube and 100 µl Trichlormethan were added and incubated at RT for additional 15 min. Samples were centrifuged for 15 min at 11 500*g* and 4°C and the upper aqueous phase was transferred to a new tube. For purification of the total RNA from the aqueous phase, we used the SV Total RNA Isolation System, Promega (Promega GmbH, Mannheim, Germany) according to the manufacturer's protocol, which included a DNase digestion step. After purification, RNA concentration was measured using NanoPhotometer Pearl (Implen, München, Germany) according to the manufacturer's instruction. A measure of 100 ng of purified total RNA from each sample were used in reverse transcription using the SuperScript III (Invitrogen by Life Technologies GmbH, Darmstadt, Germany) with random hexamer primers according to the manufacturer's protocol. Resulting cDNA was used undiluted for quantitative PCR (qPCR) analysis using gene-specific primers (table 1), which were designed in a way that they span an exon–exon boundary where possible. Amplification efficiencies (*E*) of primers were determined with five dilutions (undiluted, 1 : 10, 1 : 100, 1 : 1000, 1 : 10 000) of template cDNA, where *E* = 10–1/slope. For qPCR reaction we used the Kapa Sybr Fast qPCR Mastermix for LightCycler480 (peqlab Biotechnologie GmbH, Erlangen, Germany), according to the manufacturer's instructions. Resulting crossing point (Cp) values were calculated with LightCycler480 software using second derivative maximum method [48]. For all technical replicates, the mean Cp and the standard deviation (s.d.) were calculated. As reference we used the geometric mean of the two housekeeping genes *rp49* [49] and *rpL13a* [50]. Figure 4 is based on the relative fold expression differences (rE) between treatment groups and control groups, calculated according to Pfaffl [51].

**Table 1.** Primer sequences for qRT-PCR analysis in *T. castaneum*.

gene	Gen ID	forward primer 5–3	reverse primer 5–3
*attacin*	100141947	caaacgaccaaagggaaacta	cttccaagcaaagttgg
*coleoptericin*	100359370	tttggcactttttgcacttg	gggatgtcctgttctacgga
*GNBP*	660764	attgaccgacttcatgaccaag	agtccaacggccgtgtttag
*Hsp68*	663293	cctattcctgcgtcggagtc	ggcaacttggttcttggcag
*hsp90*	656270	cgcagttcattggctatccc	gtcttcgccttcttcctcct
*Imd*	660509	cctccaagggatgaagtcaa	actggcaaaagcagatggtc
*lysozyme*	658610	aatgcctgggctgtgtatcc	tgctttaacatcgtcaacatcagg
*nimB*	658264	cacaagggaatgggaccagg	tgccattagggcagccattt
*PGRP*	660982	ccgcgtcaaaggcattcaaa	tgccatcacccccaatcaag
*proPO*	641512	gctcaaggacccccattgaa	gaagggatacaccaggtgcc
*rp49*	658058	ttatggcaaactcaaacgcaac	ggtagcatgtgcttcgttttg
*rpL13a*	663151	ggccgcaagttctgtcac	ggtgaatggagccacttgtt
*thaumatin*	663483	atggttgctatcgagccgc	aaccccgttgccatttctga
*Toll*	656158	tcagacggaaatgcaccttg	gcaactgcaacacctcaagc

### (d) Statistics

All statistical analyses were performed in JMP 10 (SAS Institute Inc., USA).

In order to test for differences of survival rates between priming treatments only the dataset of *Bt* challenged animals in the F_1_ generation was used. For survival, a generalized linear model was applied with priming (none, genetic, step, both), line of male or offspring and mating order as factors. The response variable was the state of the animals (dead, alive) at the end of the experiment (day 7) using a binomial error distribution. *Post hoc* a contrast test was used. Offspring survival was neither affected by line of the male, nor by the line of offspring.

PO measurements were Box–Cox-transformed to achieve normal distribution. Differences in PO activity were thereafter analysed in an ANOVA with priming or mating order as well as line of male or offspring serving as factors. Tukey HSD *post hoc* test was used subsequently. PO activity was neither affected by line of the male, nor by the line of offspring.

Total number of offspring of each female was collected by counting all offspring of each batch. To test for differences in fecundity, an ANOVA was carried out with priming order, and line of male served as factors. The response variable was fecundity. A Tukey HSD *post hoc* test was used subsequently.

As the proportions of offspring sired by the first (P_1_) and second male (P_2_) regarding mating sequence are mathematically related (P_2_ = 1 − P_1_), data analysis was performed only on the proportion of offspring sired by the second male (P_2_). We calculated this proportion as expected under a binomial distribution where the expected proportion of each offspring being sired by the second male equalled 0.5 for each pair and carried out an ANOVA with priming and time as factors. The response variable was P_2_.

The variance of expression levels of each gene was calculated with Relative Expression Software Tool (REST 2009, [52]), based on the primer efficiencies and overall maximal/minimal Cp values providing the variance of each primer product across all samples and a pairwise comparison against the control treatment. To correct for multiple comparisons, we used the false discovery rate (Benjamini Hochberg correction [53]). The expression of immune and stress genes were neither affected by line of the male, nor by the line of offspring. The expression of the two housekeeping genes *rpl49* and *rpL13a* was stable across all treatments and served as control. We have tested the stability of the chosen housekeeping genes using geNorm [54]. As the *M*-value describes the variation of a gene compared to all other candidate genes, our pair of housekeeping genes is recommended as the optimum of reference genes (*rpl49 M*-value: 0.29; *rpL13a M*-value: 0.29).

## 3. Results

### (a) Survival upon bacterial challenge

**Figure 2.**
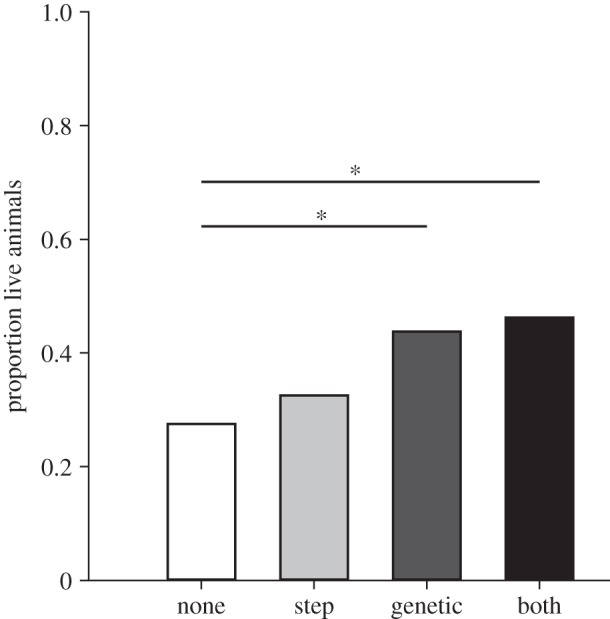
Result of a bacterial challenge in *T. castaneum* offspring after paternal immune priming. One offspring per father was either randomly assigned to bacterial challenge, to sham treatment with PBS, or left naive as control. Each treatment group consisted of 40 individuals (*n* = 40). Asterisks show significantly different survival rates between the priming treatments.

Bacterial challenge of offspring with *Bt* lead to average survival rates between 27 and 47% for the different treatments (figure 2), whereas none of the naive control animals and only 1.25% of the sham treated animals died. Priming did significantly affect the survival of the offspring (generalized linear model (GLM), *χ*^2^_3_ = 8.401; *p*_3_ = 0.038). To analyse whether paternal immune priming affected only the genetic offspring or also the step offspring resulting from the double mating, all challenged offspring were classified according to their genetic relationship to the primed male, which was accordingly denoted as ‘genetic’ or ‘step’. Offspring of fathers that were primed with heat-killed bacteria showed a significantly better survival than offspring of non-primed fathers (GLM, *χ*^2^_3_ = 4.694; contrast: *p*_3_ = 0.03). Priming of stepfathers, however, did not provide a survival benefit (GLM, *χ*^2^_3_ = 0.482; contrast: *p*_3_ = 0.487). When both father and stepfather were primed, offspring survival was also significantly enhanced (GLM, *χ*^2^_3_ = 6.168; contrast: *p*_3_ = 0.013).

### (b) Phenoloxidase activity

**Figure 3.**
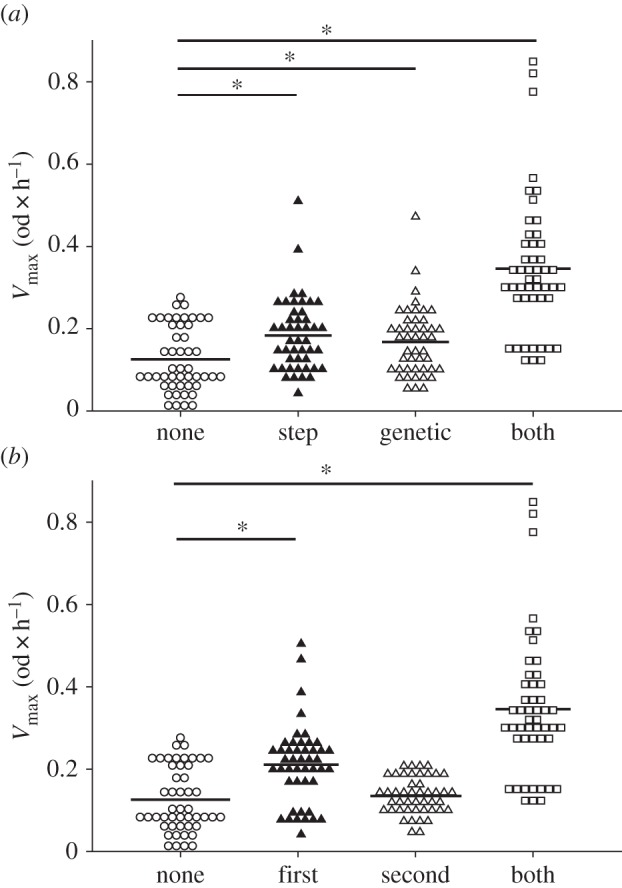
Constitutive PO measurement of haemolymph samples taken from naive offspring. (*a*) Offspring is categorized by the relationship to the primed male. None (*n* = 48); step (*n* = 46); genetic (*n* = 46); both (*n* = 48). (*b*) Offspring is categorized by mating order. None (*n* = 48); first (*n* = 46); second (*n* = 46); both (*n* = 48). Asterisks show significantly different PO between the priming treatments.

Constitutive PO of offspring was significantly increased when any of the two males a female was mated to had received immune priming, as compared with non-primed males (ANOVA, *F*-ratio = 36.421; d.f. = 3; *p*_3_ = 0.0001; figure 3*a*). In contrast to the effect on offspring survival, this effect was independent of the relationship of the offspring to the male that was primed, that is, father and stepfather were equally relevant. However, the mating order had an effect on PO of the offspring (ANOVA, *F*-ratio = 44.32; d.f. = 3; *p*_3_ = 0.0001). If the first male was primed, all the offspring of the double-mating set show an elevated PO compared with offspring of double-mating sets where the second male was primed. Furthermore, our data indicate that the effect was additive, because PO of offspring derived from double-mating sets where both males were primed was highest (figure 3*b*).

### (c) Immune and stress gene expression

**Figure 4.**
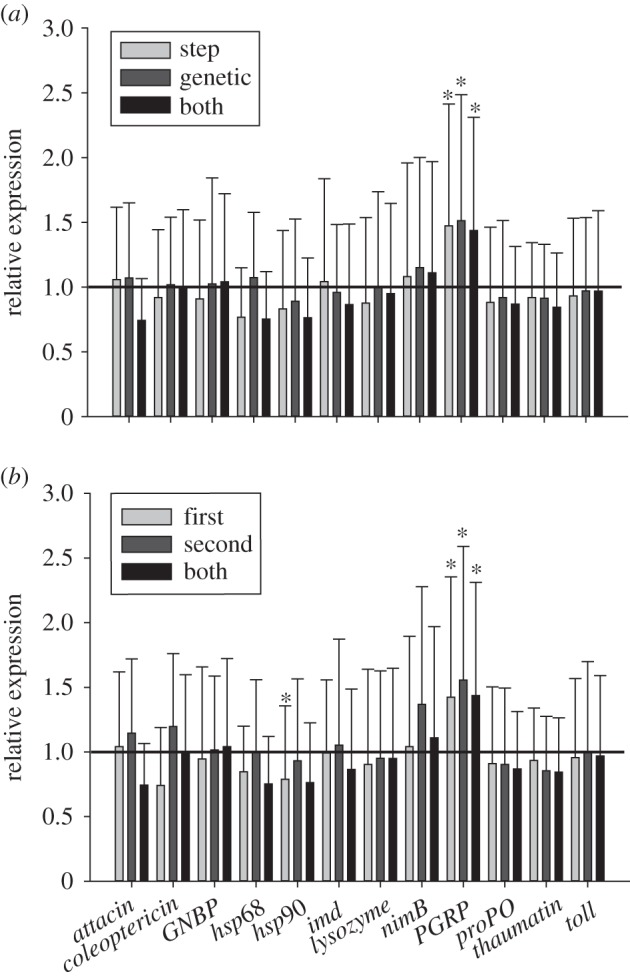
Constitutive relative gene expression measurements from naive offspring. (*a*) Offspring is categorized by the relation to the primed male. (*b*) Offspring is categorized by the mating order. Each treatment group consisted of 12 pools (*n* = 12).

The expression of most of the immune and stress genes studied here was not significantly affected by paternal priming (electronic supplementary material, table S1). However, paternal immune priming significantly affected the immune gene peptidoglycan recognition protein (*PGRP*). All treatments i.e. both (mean expression: 1.437; *p* = 0.044), genetic (mean expression: 1.513; *p* = 0.028) and step (mean expression: 1.473; *p* = 0.026) priming showed an increased relative expression compared with the control treatment, i.e. father and stepfather priming similarly increased offspring PGRP expression, and there was no further increase when both males had been primed. Moreover, insignificant trends were found for an upregulation of the phagocytosis receptor *nimB* and for a downregulation of the stress related gene *hsp90* (figure 4).

Mating order did not seem to be relevant for the relative expression of the immune and stress related genes. *PGRP* was significantly upregulated irrespective of mating order. *Hsp90* showed a trend for downregulation for all mating orders, but this effect was significant only when the first male was primed (mean expression: 0.789; *p* = 0.031).

### (d) Total number of offspring

Priming of males had a significant effect on female reproduction (ANOVA, *F*-ratio = 3.481; d.f. = 3; *p*_3_ = 0.017). Females that were mated to two naive males produced fewer offspring than females that mated with two primed males. When one of the males was primed, offspring numbers were intermediate, irrespective of whether the primed male was the first or second partner.

### (e) Paternity

The proportion of offspring sired by the second male (P_2_) was not affected by the priming treatments (ANOVA, *F*-ratio = 1.043; d.f. = 3; *p*_3_ = 0.375). Irrespective of the priming, P_2_ is around 80% in the first batch of offspring and decreases continuously to *ca* 55% in the last batch of offspring 10 days after mating. However, P_2_ differed slightly between the beetle lines, such that Cro1 had on average a P_2_ ratio that was 5% higher than the reindeer line (ANOVA, *F*-ratio = 51.536; d.f. = 1; *p*_1_ = 0.0001).

## 4. Discussion

Previous studies of trans-generational effects on insect immunity have concentrated on expression of immune system components in the offspring as a consequence of stimulation of the parental immune system [6–10,18,21,23,24,26,55,56]. The first study of paternally transmitted resistance by Roth *et al.* [26] demonstrated that fathers also pass on resistance to their offspring. Our study confirms these findings and provides information in which way such transfer might occur. Using a double-mating set-up, we were able to show that an increased survival after challenge correlates with the relationship of the offspring to the primed male. Offspring related to the primed male show increased survival rates whereas step offspring sired by the same female mated with a non-primed male show a survival rate that is not significantly different from naive offspring. Offspring survival was similarly increased when both males were primed.

In theory, the transfer of an induced phenotype from parents to offspring could be accompanied by a modification of genetic material in the germ line either via stable genetic alteration (e.g. mutation, change in the DNA sequence) or via epigenetic mechanisms (e.g. DNA methylation or histone acetylation) [57]. By definition, epigenetic effects describe the variance in offspring phenotype without alteration of the DNA sequence, being triggered by experiences such as environmental changes, density, diet, chemical exposure and pathogens [10,23,24,26,27,45,58–60]. Possible mechanisms are, for example, DNA methylation, histone modification, chromatin remodelling and DNA silencing via non-coding RNA [57]. Our survival data suggest that the male germ line is responsible for the transmission of the trans-generational phenotype. From an evolutionary point of view, trans-generational effects like paternal immune priming might have three considerable advantages on phenotypic evolution: (i) individually acquired traits in life, such as a primed immune system, can be directly transmitted to the offspring, (ii) an opportune pool of traits can be transferred over generations, and therefore (iii) a response to selection pressure occurs in the short term [61].

Our results obtained for haemolymph PO as a general but fast innate immune component for infection but also wounding [26] reveals a more complicated picture of paternal trans-generational immunity. In our study, offspring of immune primed males show an elevated PO as observed before in our laboratory [26] and in a study on the mealworm beetle, *T. molitor* [11]. Indeed, in this study all offspring, independent of the relationship to the primed father, show an elevated PO compared with control offspring. Measuring constitutive PO levels of offspring, we found no differences between offspring sired by the primed male and the step offspring. This might indicate a paternal priming effect transferred via seminal fluid. Male ejaculates are complex and comprise more than just sperm. Produced by secretory tissues in the reproductive tract, it can include seminal fluid proteins, salts and sugars, defensive compounds and lipids [29]. Recent work in *T. castaneum* has identified several specific non-sperm components of male ejaculates [62]. While seminal fluid proteins assist in storing and provisioning sperm [29], they also show immune stimulatory responses in insects [28]. Thus, it is conceivable that transferring this complex cocktail together with the sperm to a female during mating may lead directly to paternal trans-generational effects via immune stimulatory compounds like antimicrobial peptides [63]. On the other hand, the effect on offspring might also be indirect via manipulation of the female. Seminal fluid proteins might indirectly influence the female [64] by modifying epigenetic factors [65], such as regulatory RNA that affect the expression of immune genes [66,67] or immunostimulatory proteins [29] in the next generation. Furthermore, the paternal transfer of induced PO seems to be additive when both males in the double-mating set-up are primed and implies a dosage dependency of the triggering component in the ejaculate.

In more detail, we found evidence that not only priming but also mating order of the primed male had an effect on the constitutive PO level of offspring. Possibly, the reproductive state of the female plays a role. If the female was mated before with a naive male the effect of paternal trans-generational immune priming is less prominent than in the case of a virgin female mated with an immune primed male first. In *T. molitor*, it was shown that only the offspring of early reproductive episodes showed paternal trans-generational immune priming [11]. It appears that the mated female which already stored sperm to fertilize eggs is less affected by paternal immune priming.

The results for the relative expression of immune and stress-related genes picture a general alertness that is transferred via paternal immune priming. We find an upregulation of the gene *PGRP* in all three paternal priming treatments and independent of mating order. PGRPs are innate immunity molecules present in insects, molluscs, echinoderms and vertebrates, but not in nematodes or plants. In insects, they are present in the haemolymph, cuticle and fat-body cells, and sometimes in epidermal cells in the gut and haemocytes. The expression of insect PGRPs is often upregulated by exposure to bacteria to protect against infection. They activate the Toll or Imd signal transduction pathways or induce proteolytic cascades that generate antimicrobial products, induce phagocytosis, or hydrolyze peptidoglycan [68]. In this case, *PGRP* stimulates the activation of the Imd pathway leading to synthesis of antimicrobial peptides in response to DAP-type peptidoglycan. In *Drosophila*, it was shown that DAP-containing peptidoglycan extracted from the Gram-positive *Bt* induced the Imd pathway via *PGRP* [69]. Furthermore, it was shown that PGRP controls the melanization cascades in *Drosophila* [70]. The melanization cascade mediates haemolymph activation, wound clotting, and melanin production at the exoskeletal breakage site to prevent internal spread of microorganisms [71,72]. Melanization is dependent on PO.

While we find an effect on the expression of the immune receptor PGRP, we do not find an effect on transcriptional levels of the immune effector proPO. proPO is transferred into active PO upon enzymatic cleavage, which is a tightly regulated process. Our enzymatic PO assay showed an effect of paternal immune priming. We thus hypothesize that gene expression in offspring at the time-point where we measured it was unchanged, but higher levels of the proPO enzyme in the haemolymph might result in higher enzymatic activity upon activation during haemolymph withdrawal. In addition, the higher levels of PGRP might also contribute to an increased sensitivity towards activation of the PO cascade. Taken together, these results suggest that paternal immune priming leads to a higher level of immunological alertness of offspring.

To check for effects of paternal immune priming related to phagocytosis we quantified expression of the gene *nimB* belonging to the Nimrod superfamily [73]. Several members of the superfamily were claimed to function as receptors in phagocytosis or binding of bacteria, which indicates an important role in the cellular immunity. Interestingly, they have been identified only in insects so far [74]. We find a trend for the involvement of *nimB* in paternal immune priming. In both treatments, *nimB* showed a tendency for upregulation compared with the controls. Furthermore, our results for paternal immune priming show a trend for reduced expression of *hsp90*. Beside numerous functions of *hsp90* in cellular processes and developmental pathways [75], *hsp90* might also be involved in trans-generational immune priming. Reduced *hsp90* expression could result in increased phenotypic variation, and thereby enhanced evolvability [76,77]. This could be beneficial in stressful environments such as under parasite pressure. However, this hypothesis needs further investigation. As this was, to our knowledge, the first attempt to measure immune gene expression of the offspring after paternal trans-generational immune priming in insects, the next step will be to detect differences in expression of the genes in the offspring over time after an immune challenge. This more extensive approach would be interesting because differences might be visible in the kinetics, or in a distinctive time window.

As priming may affect fecundity and differences of paternity success in our double-mating experiment, we measured fecundity and calculated P_2_ [4]. In this study, P_2_ was not affected by paternal immune priming. The beetle line differences in P_2_ we found might be owing to the higher inbreeding of the reindeer line. Moreover, the number of offspring produced in the early reproduction that was measured here is increased if the female mated with two primed males. Females might shift towards earlier reproduction when mated to primed males. Moreover, as these males’ immune status is elevated after priming they might be more attractive to the female. In *T. molitor*, it was suggested that pheromones indicate the immunocompetence of the male and alter the pre-mating behaviour of the female resulting in higher fecundity [78].

In summary, our study sketches a complex picture of paternal trans-generational immune priming, indicating that the information for better survival of offspring is transferred via the father, whereas the trigger for elevated PO and for immune receptor gene expression seems to be influenced via the seminal fluid. To what extent these substances affect offspring directly or indirectly via the female needs further investigation.

As hypothesized by Roth *et al.* [26] paternal immune priming seems to be more general, implicating the innate immune system, while mothers transfer a more specific immune response [30]. As a matter of fact both sexes are in charge of protecting their offspring against a parasitic environment that they have encountered themselves. Therefore, these findings provide new insights into the field of ecological immunity with potentially relevant ramifications for host–parasite coevolution and sexual selection [30].

## Supplementary Material

Statistical analysis of gene expression data
