# Supplementary material for: Different effects of paternal trans-generational immune priming on survival and immunity in step and genetic offspring
Source: Proc Biol Sci. 2014 Dec 22;281(1797):20142089. doi: 10.1098/rspb.2014.2089 (PMC4240996; doi:10.1098/rspb.2014.2089)
Supplement: Statistical analysis of gene expression data [file rspb20142089supp1.pdf]

Tab. 4: Statistical analysis of gene expression data. The variance of expression levels of each gene was calculated with Relative Expression Software Tool (REST 2009), based on the primer efficiencies and overall maximal/minimal Ct values providing the variance of each primer product across all samples and a pairwise comparisons against the control treatment. To correct for multiple comparisons we used the false discovery rate (FDR);Benjamini Hochberg. Shown are the effects of the factors (a) priming (none, step, genetic, both) and (b) mating order (none, second, first, both). * = significant after FDR

Gene	treatment	mean expression	*P*	treatment	mean expression	*P*
attacin	stepfather	1,058	n.s.	first	1,04	n.s.
attacin	father	1,071	n.s.	second	1,145	n.s.
attacin	both	0,743	n.s.	both	0,743	n.s.
coleoptericin	stepfather	0,918	n.s.	first	0,945	n.s.
coleoptericin	father	1,017	n.s.	second	1,015	n.s.
coleoptericin	both	1,002	n.s.	both	1,04	n.s.
GNBP	stepfather	0,908	n.s.	first	0,741	n.s.
GNBP	father	1,024	n.s.	second	1,197	n.s.
GNBP	both	1,04	n.s.	both	1,002	n.s.
hsp68	stepfather	0,767	n.s.	first	0,846	n.s.
hsp68	father	1,072	n.s.	second	1,003	n.s.
hsp68	both	0,753	n.s.	both	0,753	n.s.
hsp90	stepfather	0,831	n.s.	first	0,789	0,031
hsp90	father	0,89	n.s.	second	0,931	n.s.
hsp90	both	0,763	n.s.	both	0,763	n.s.
imd	stepfather	1,042	n.s.	first	0,992	n.s.
imd	father	0,958	n.s.	second	1,053	n.s.
imd	both	0,865	n.s.	both	0,865	n.s.
lysozyme	stepfather	0,877	n.s.	first	0,903	n.s.
lysozyme	father	0,996	n.s.	second	0,951	n.s.
lysozyme	both	0,95	n.s.	both	0,95	n.s.
nimB	stepfather	1,08	n.s.	first	1,041	n.s.
nimB	father	1,15	n.s.	second	1,368	n.s.
nimB	both	1,11	n.s.	both	1,11	n.s.
PGRP	stepfather	1,473	0,026	first	1,424	0,024
PGRP	father	1,513	0,028	second	1,557	0,012
PGRP	both	1,437	0,044	both	1,437	0,037
proPO	stepfather	0,881	n.s.	first	0,91	n.s.
proPO	father	0,919	n.s.	second	0,903	n.s.
proPO	both	0,868	n.s.	both	0,868	n.s.

thaumatin	stepfather	0,918	n.s.	first	0,934	n.s.
thaumatin	father	0,913	n.s.	second	0,855	n.s.
thaumatin	both	0,844	n.s.	both	0,844	n.s.
toll	stepfather	0,932	n.s.	first	0,956	n.s.
toll	father	0,97	n.s.	second	1,004	n.s.
toll	both	0,968	n.s.	both	0,968	n.s.
